# Regulation of ErbB2 localization and function in breast cancer cells by ERM proteins

**DOI:** 10.18632/oncotarget.8327

**Published:** 2016-03-23

**Authors:** Nagham Asp, Audun Kvalvaag, Kirsten Sandvig, Sascha Pust

**Affiliations:** ^1^ Department of Molecular Cell Biology, Institute for Cancer Research, Oslo University Hospital, 0379 Oslo, Norway; ^2^ Centre for Cancer Biomedicine, Faculty of Medicine, University of Oslo, 0379 Oslo, Norway; ^3^ Current address: Department of Molecular Medicine, Division of Biochemistry, University of Oslo, 0379 Oslo, Norway; ^4^ Department of Molecular Biosciences, Faculty of Mathematics and Natural Sciences, University of Oslo, 0379 Oslo, Norway

**Keywords:** ezrin, radixin, breast cancer, ErbB receptor tyrosine kinsases

## Abstract

The ERM protein family is implicated in processes such as signal transduction, protein trafficking, cell proliferation and migration. Consequently, dysregulation of ERM proteins has been described to correlate with carcinogenesis of different cancer types. However, the underlying mechanisms are poorly understood. Here, we demonstrate a novel functional interaction between ERM proteins and the ErbB2 receptor tyrosine kinase in breast cancer cells. We show that the ERM proteins ezrin and radixin are associated with ErbB2 receptors at the plasma membrane, and depletion or functional inhibition of ERM proteins destabilizes the interaction of ErbB2 with ErbB3, Hsp90 and Ebp50. Accompanied by the dissociation of this protein complex, binding of ErbB2 to the ubiquitin-ligase c-Cbl is increased, and ErbB2 becomes dephosphorylated, ubiquitinated and internalized. Furthermore, signaling via Akt- and Erk-mediated pathways is impaired upon ERM inhibition. Finally, interference with ERM functionality leads to receptor degradation and reduced cellular levels of ErbB2 and ErbB3 receptors in breast cancer cells.

## INTRODUCTION

The members of the ERM protein family (ezrin, radixin and moesin) are scaffolding proteins at the plasma membrane and act as functional linkers between the membrane and the actin cytoskeleton [1]. The prominent localization of ERM proteins at the plasma membrane is also reflected by their structural and functional involvement in a multitude of cellular processes. Originally, ERM proteins have been described as structural components necessary for actin organization, and they are involved in processes such as sustaining of cell morphology and cell migration [2–4]. However, it became evident that ERM proteins play also an important role in signal transduction. Hence, activation of ERM proteins by binding to PI(4, 5) P_2_ and phosphorylation has been shown to be a prerequisite for activation of different signaling pathways [5–7]. Moreover, the interaction between ERM proteins and transmembrane receptors, including receptor tyrosine kinases (RTKs), is required for various signal transduction pathways [8]. During the last decade new functions of ERM proteins in membrane- and protein-trafficking have been elucidated. Thus, members of the ERM protein family have been shown to regulate protein sorting, recycling and retrograde transport [9–12]. Beside the regulation of physiological processes, ERM proteins are also involved in pathological processes, such as tumor progression of different cancer types [8]. In particular, activation of ERM proteins triggers invasion and metastasis of breast cancer, and increased expression and abnormal distribution of ezrin have been associated with poor prognosis for breast cancer patients [13–15]. ERM proteins have been described to be associated with ErbB receptor tyrosine kinases (RTKs) and are considered to have a regulatory role in RTK function [16–19]. Interestingly, ezrin has been found in a molecular complex with CD44, Hsp90 and ErbB2 in mammary carcinoma cells [20]. However, the mechanistic function of ERM proteins in the regulation of ErbB2 receptor tyrosine kinases and carcinogenesis is not yet understood.

ErbB receptor tyrosine kinases (EGFR/ErbB1, ErbB2, ErbB3 and ErbB4) are known to activate cellular transformation, migration, and proliferation [21, 22], and they are also implicated in development and progression of several cancer types (reviewed in [23]). In particular, amplification and overexpression of ErbB2 and ErbB3 have been correlated to poor prognosis for breast cancer patients [24, 25]. The ErbB2-ErbB3 heterodimer contains high oncogenic potential by constitutive activation of several signaling cascades [21]. ErbB dimerization leads to the activation of Akt- and Erk-dependent signal transduction pathways. Both pathways are known to stimulate cell growth and survival. Consequently, overstimulation and continuous signaling through these pathways is a driving mechanism for carcinogenesis [26, 27]. Thus, ErbB receptors have become attractive targets for anti cancer therapies and several ErbB targeted therapies have been developed for clinical use [21, 28, 29]. However, many patients do not respond or develop drug-resistant cancers after treatment with ErbB targeting agents [23, 30, 31]. This emphasizes the need to understand the underlying mechanisms of ErbB mediated carcinogenesis to develop new anti-cancer strategies. Beside the significance of ErbB receptors in the activation of Akt/Erk signaling, also ERM proteins are known to interact with several components of these pathways or with transmembrane receptors that are upstream of these cascades [8]. For example, ERM proteins can regulate the activity of Ras [32–34], which itself is crucial for the activation of the Erk pathway. Therefore, ERM proteins can directly modulate oncogenic signaling pathways and in many clinical studies ERM overexpression have been linked to tumourigenesis and poor outcome in cancer patients [8].

Here, we report a novel functional interplay between ERM proteins and ErbB2 receptors in breast cancer cells. ERM proteins are essential components of a multiprotein complex including ErbB2, ErbB3, Hsp90, and Ebp50. Depletion of ezrin or radixin triggers the dissociation of this complex, in addition to significant association of c-Cbl to ErbB2, ErbB2 ubiquitination and dephosphorylation. Furthermore, depletion or functional inhibition of ezrin or radixin leads to relocalization of ErbB2 and ErbB3 receptors to intracellular vesicles and reduced cellular ErbB2/3 levels. As a consequence, ErbB2- and ErbB3-induced signaling via Akt- and Erk-dependent pathways is reduced. Thus, our data demonstrates a regulatory role for ERM proteins in membrane localization, complex stabilization of ErbB2 receptors and oncogenic downstream signaling.

## RESULTS

### The ERM proteins ezrin and radixin are associated with ErbB receptors at the plasma membrane in breast cancer cells

**Figure 1:**
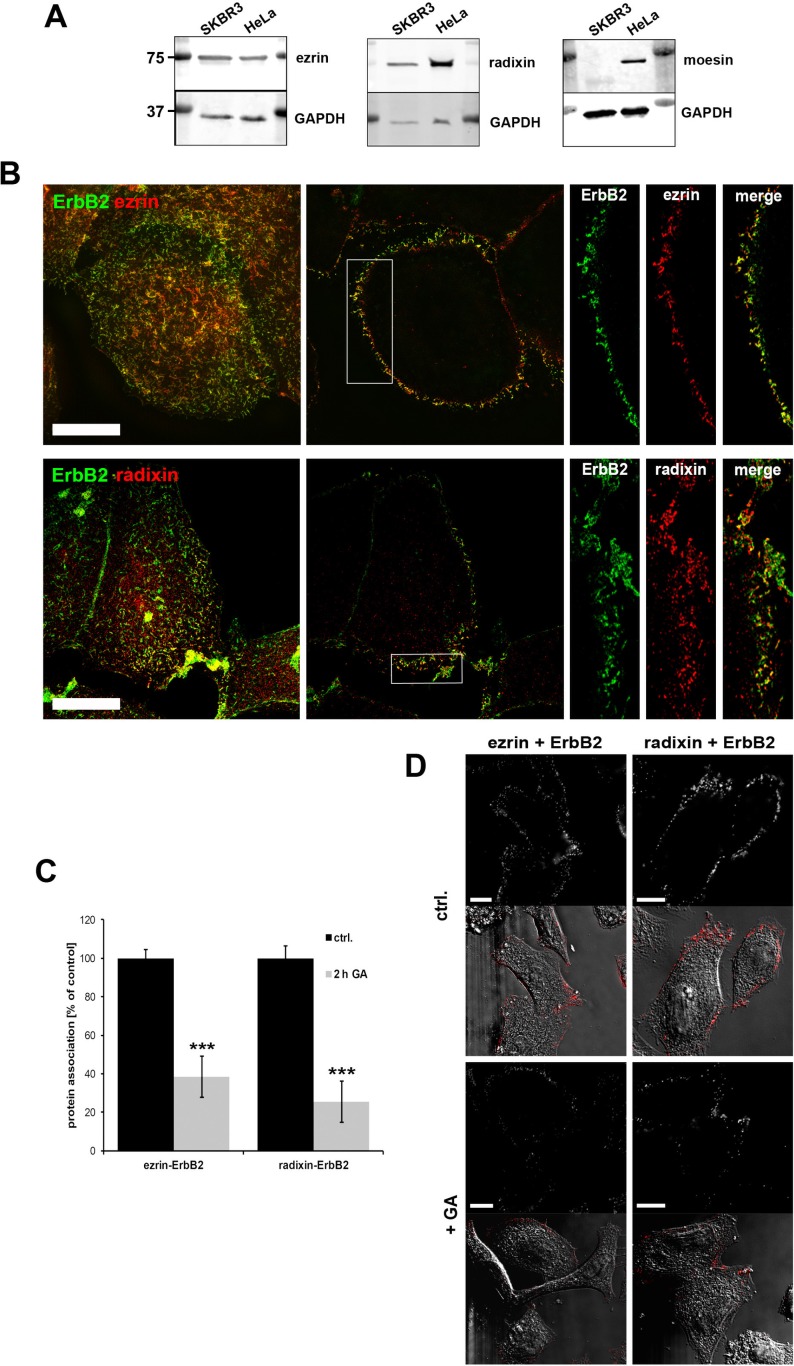
ERM expression and localization in SKBR3 breast cancer cells (**A**) Western blot analysis of ERM levels in SKBR3, HeLa and PC3 cells. SKBR3 breast cancer cells do not express moesin. (**B**) Colocalization of ezrin/radixin and ErbB2 in SKBR3 cells. 3D-SIM of fixed cells, stained for endogenous ERM and ErbB2, shows a high degree of colocalization between the ezrin/radixin and ErbB2 at the plasma membrane (left panel: max. projection; middle: single plane section; right: single channels of insert). Scale bars: 10 μm. (**C**) Analysis of protein association in SKBR3 cells by proximity ligation assay (PLA). 2 h treatment with 3 μM GA leads to decreased association of ezrin/ErbB2 and radixin/ErbB2. Data is represented as mean +/− SEM (***P < 0.001). (**D**) Corresponding single plan section of a representative PLA experiment. Fluorescence and DIC pictures of control cells (upper panel) and cell treated for 2 h with geldanamycin (lower panel) are shown. Scale bars: 10 μm.

In a first step, we checked the protein levels of the single ERM proteins in SKBR3 breast cancer cells, which express high levels of ErbB2. In contrast to HeLa cells, SKBR3 cells showed no expression of moesin (Figure 1A), and we therefore focused on the presumed role of ezrin and radixin in the regulation of ErbB2 receptors in this breast cancer cell line. According to this, we performed cellular localization studies of ezrin, radixin and ErbB2 in SKBR3 cells. By 3D-SIM analysis we obtained a clear colocalization of ezrin/radixin with ErbB2 (Figure 1B). Ezrin and radixin were colocalized with ErbB2 at the plasma membrane, particularly in actin-enriched and lamellipodia-like structures. It has been shown that ErbB2 preferentially heterodimerize with ErbB3 [35] and this represents the predominant ErbB heterodimer in this cell line. Accordingly, also colocalization of ezrin and radixin with fluorescently labled CFP-ErbB3 (Supplementary Figure 1A) or endogenous ErbB3 (Supplementary Figure 1B, upper panel) was observed at the plasma membrane. The colocalization data were supported by findings from proximity ligation assays (PLA), indicating an interaction of ERM proteins and ErbB2 within a proximity of < 40 nm (Figure 1C and 1D, control). In order to examine the proximity of the ERM-ErbB2 interaction at even higher resolution, we performed FRET (fluorescence resonance energy transfer) acceptor photobleaching experiments on fixed cells [36]. By using Alexa488- labeled antibodies against ezrin as donor and Alexa568-labeled antibodies against ErbB2 as acceptor (Supplementary Figure 1C), we obtained a FRET efficiency of 8.8 +/− 2.8%. Thus, our data demonstrate close spatial proximity between ezrin/radixin and ErbB2 receptors at the plasma membrane.

### Depletion or inhibition of ezrin/radixin leads to reduced ErbB2 and ErbB3 protein levels

**Figure 2:**
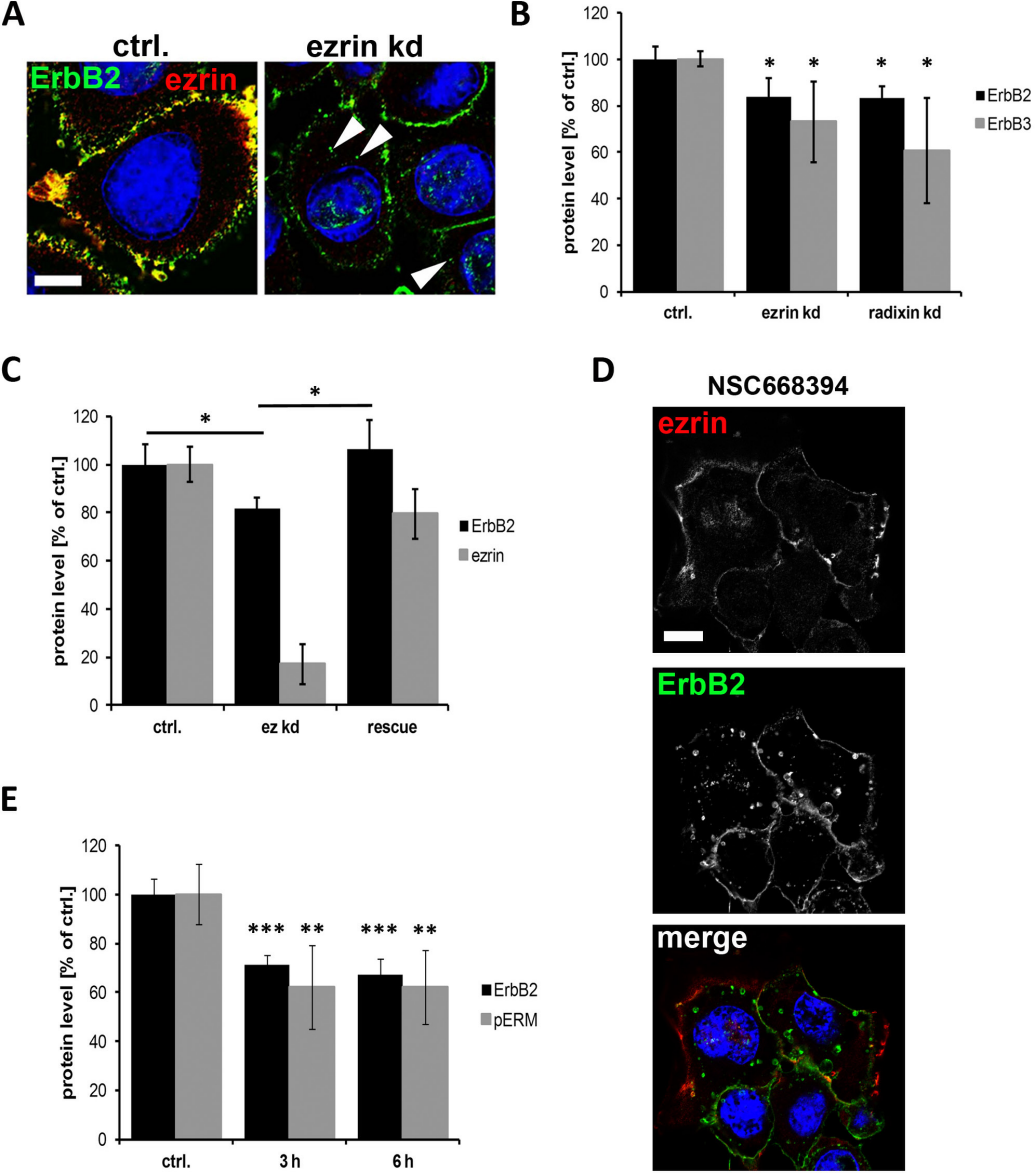
Internalization and degradation of ErbB receptors after interference with ERM proteins (**A**) Localization of ErbB2 in control and ezrin depleted SKBR3 cells. As observed by confocal microscopy (single plane section), ezrin depletion leads to localization of ErbB2 in intracellular vesicles (arrowheads). Scale bars: 10 μm. (**B**) Quantification of Western blot analysis of ErbB2 and ErbB3 protein levels after ERM knockdown. Depletion of ezrin or radixin leads to significantly reduced protein levels of ErbB2 and ErbB3. (**C**) ErbB2 protein level after rescue of ezrin levels. Cells rescued for ezrin levels by transfection of a siRNA resistant ezrin DNA upon ezrin knockdown, leads to restored protein levels of ErbB2. (**D**) Confocal microscopy (single plane section) of ErbB2 localization. Inactivation of ERM proteins by NSC668395 (3 h) leads to internalization of ErbB2 into vesicular structures. Scale bars: 10 μm. (**E**) Quantification of Western blot analysis of ErbB2 and pERM levels after treatment with NSC668394 for 3 h and 6 h. All data in this Figure represented as mean +/− SEM (*P < 0.05; **P < 0.01; ***P < 0.001).

It has been demonstrated earlier that internalization and subsequent degradation of ErbB2 and ErbB3 receptors can be induced either by GA treatment [37] or by knockdown of the ErbB stabilizing flotillin proteins [38, 39]. To investigate whether also ERM proteins stabilize the level of ErbB receptors at the membrane, we first analyzed the effect of ERM depletion by siRNA on the localization and the protein levels of ErbB2 and ErbB3. Interestingly, knockdown of ezrin or radixin (Supplementary Figure 1D and 1E) induced the accumulation of ErbB2 in intracellular vesicles, as shown in Figure 2A. Moreover, ErbB2 and ErbB3 levels were 20–40% reduced upon depletion of ezrin or radixin (Figure 2B and Supplementary Figure 1D). Conversely, restoring ezrin protein levels by transfection of a siRNA resistant ezrin construct led to a complete rescue of ErbB2 levels (Figure 2C). In addition to protein depletion we used the inhibitor NSC668394 to functionally inhibit ERM proteins. This inhibitor has been described to interfere with ERM phosphorylation and thereby lead to impaired functional activity of these proteins [40]. Similar to depletion of ERM proteins, we obtained the appearance of internalized ErbB2 receptors in SKBR3 breast cancer cells after treatment with NSC668394 (Figure 2D and Supplementary Figure 2A). Moreover, in response to decreased levels of phosphorylated ERM proteins (pERM), ErbB2 levels were ~40% reduced after treatment with NSC668394 for 3 h or 6 h (Figure 2E). Interestingly, the effects of NSC668394 on ERM phosphorylation and the levels of ErbB2 were reversed after replacement of the inhibitor with fresh medium and further incubation for 13 h (Supplementary Figure 2B). The correlation between pERM levels and ErbB2 levels shown in SKBR3 cells was also observed in MCF7 breast cancer cells, after treatment with NSC668394 (Supplementary Figure 2C). Thus, our data clearly demonstrate that the membrane localization and maintenance of ErbB2 and ErbB3 proteins levels depends on functional ERM proteins.

### ERM proteins are integral components of a multiprotein complex important for ErbB2/3 stabilization at the membrane

**Figure 3:**
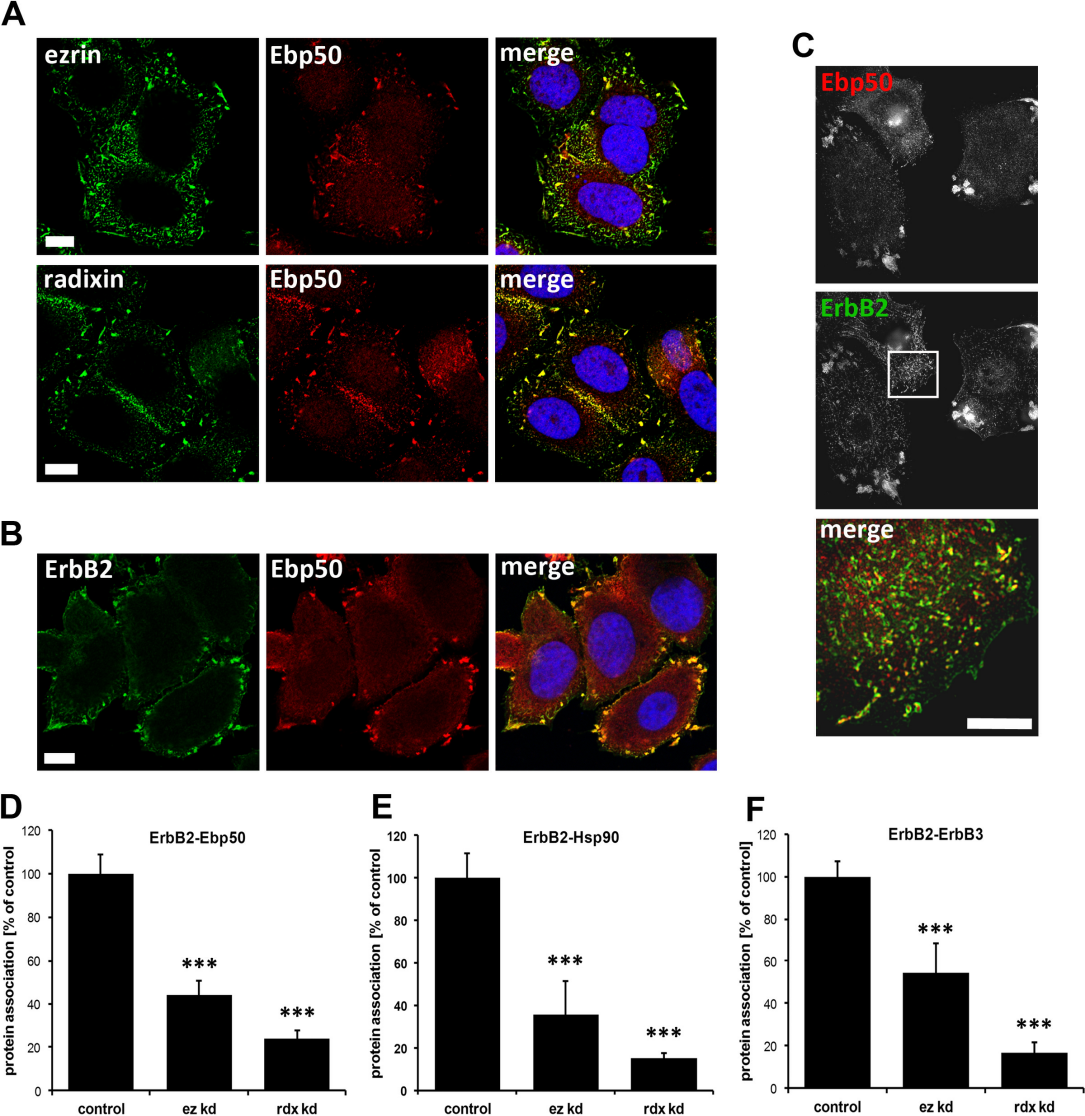
Localization studies of ERM proteins, Ebp50 and ErbB2 in SKBR3 cells and protein proximity analysis (**A**) Confocal microscopy of ERM proteins and Ebp50. Untreated cells were fixed, permeabilized and stained by specific antibodies for endogenous levels of ezrin, radixin and Ebp50 (max. projection). In SKBR3 cells ezrin and radixin strongly colocalizes with Ebp50 at the plasma membrane. (**B**) Confocal microscopy (single plane section) and (**C**) 3D-SIM (max. projections) of ErbB2 and Ebp50 localization. In untreated control conditions, ErbB2 shows a strong colocalization with Ebp50 in lamellipodia-like structures at the plasma membrane. (**D-F**) Analysis of protein proximity by PLA experiments. SKBR3 cells were transfected either with non-targeting control siRNA or ezrin/radixin specific siRNA. Cells were fixed 72 h after transfection and PLA experiments were performed. The depletion of ezrin or radixin strongly reduces the interaction of ErbB2 with Ebp50 (D), Hsp90 (E) and ErbB3 (F). All results are shown as mean +/− SEM (*P < 0.05; ***P < 0.001). Scale bars: 10 μm (A, B), 8 μm (C).

Next, we wanted to investigate the mechanisms involved in ErbB receptor degradation triggered by interference with ERM proteins. For this purpose, we studied which other proteins might be involved in the interaction between ERM proteins and ErbB2, and tested the ERM-binding phosphoprotein 50 (Ebp50/NHERF1/SLC9A3R1). Ebp50 has been demonstrated to be an important linker between membrane proteins, such as the cystic fibrosis transmembrane conductance regulator (CFTR), and ERM proteins that are connected to the actin cytoskeleton. Importantly, an interaction of Ebp50 with EGFR [41, 42] and colocalization between Ebp50 and ErbB2 in breast tissue [43] has been described earlier. In SKBR3 cells Ebp50 was colocalized with ezrin and radixin (Figure 3A), and in analogy to ERM proteins, Ebp50 was also found to be colocalized with ErbB2 at the plasma membrane, demonstrated by confocal and super resolution microscopy (Figure 3B, 3C). Furthermore, PLA experiments also revealed a close proximity between ErbB2 and Ebp50 (Figure 3D, control). In addition, we were able to verify by PLA the interaction between ErbB2 and Hsp90 (Figure 3E, control). The PLA technology was also used to show and quantify the proximity of Hsp90 and ezrin/radixin. However, the association of these proteins seems to be mediated or stabilized by ErbB2, since depletion of ErbB2 leads to a 70% reduction of ERM-Hsp90 association (Supplementary Figure 3A), without affecting the protein levels of ERM proteins, Ebp50 (Supplementary Figure 3B) or Hsp90, as demonstrated earlier [38]. Next, we analyzed by PLA the effect of ERM proteins on the interaction of ErbB2 with Ebp50, Hsp90 and dimerization of ErbB2 with ErbB3. Importantly, upon depletion of ezrin or radixin the association of ErbB2 with Ebp50 (Figure 3D), Hsp90 (Figure 3E) or ErbB3 (Figure 3F) was strongly reduced (40–80%), and there was a tendency for stronger reduction upon radixin knockdown. Therefore, we conclude that ezrin and radixin are associated to a complex including ErbB2, ErbB3, Hsp90 and Ebp50, and interference with ERM proteins seems to trigger the disruption of this complex.

### Interference with ERM proteins leads to significant association of ErbB2 with ubiquitin

**Figure 4:**
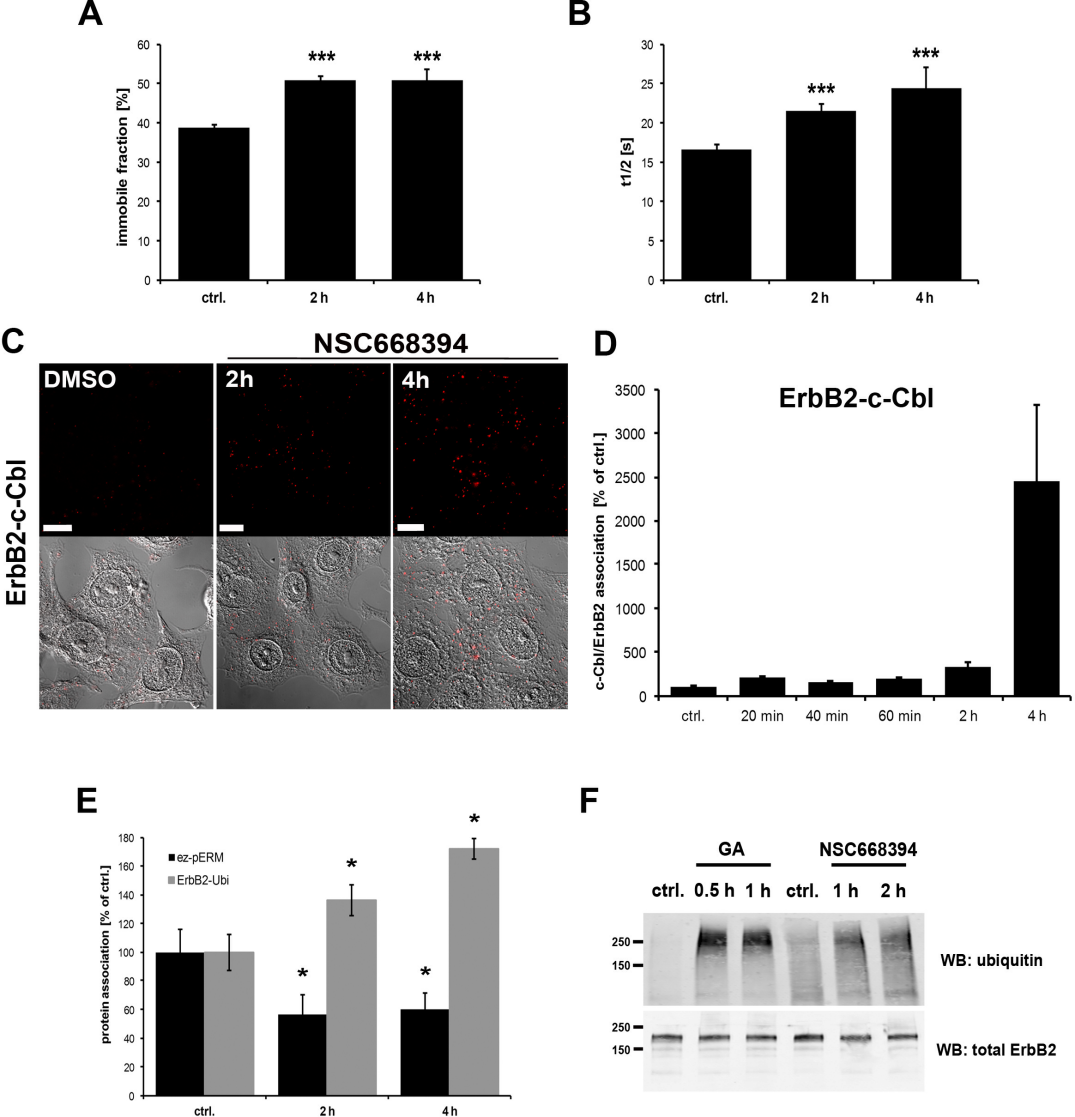
Effect of ERM inhibition on ErbB2 membrane mobility and association to ubiquitin (**A, B**) FRAP analysis of GFP-ErbB2 membrane mobility. SKBR3 cells were transfected for 16 h with GFP-ErbB2 plasmids and subsequently treated with NSC668394 for 2 h and 4 h. As described in the Methods part, specific regions were bleached and the recovery of the GFP-fluorescence was monitored and analyzed. Inhibition of ERM proteins by treatment with NSC668394 leads to a ~25% increase in the immobile ErbB2 fraction (A), accompanied by a significant increase of 20–30% in the t_1/2_ recovery time (B). (**C**) Representative confocal microscopy of ErbB2-c-Cbl PLA experiments and (**D**) quantification of ErbB2-c-Cbl PLA experiments. Cells were treated with DMSO alone or NSC668304 for the indicated periods of time and PLA experiments were subsequently performed in fixed and permeabilized cells. Inhibition of ERM proteins by NSC668394 strongly increased the association of ErbB2 with C-Cbl. Scale bars: 10 μm. (**E**) PLA experiments of ErbB2-ubiquitin association upon ERM inhibition. Cells were treated for 2 h and 4 h with NSC668394 and PLA experiments were performed. The phosphorylation status of ezrin was measured by using ezrin- and pERM- specific antibodies. Proximity of ErbB2 with ubiquitin was analyzed by the combination of ErbB2 and ubiquitin-specific antibodies. Reduction of pERM levels of 40% leads to 40–70% increase in ErbB2-ubiquitin association. All results are shown as mean +/− SEM (*P < 0.05; ***P < 0.001). (**F**) Immuoprecipitation of ErbB2-ubiquitin conjugates. Cells were treated with GA or NSC668394 or left untreated. Cell lysates were prepared and the input was probed for total ErbB2. Immunoprecipitations were performed with an anti-ErbB2 antibody. Treatment with GA as well as ERM inhibition significantly increases ubiquitination of ErbB2.

Treatment with geldanamycin, an inhibitor of head shock protein 90 (Hsp90), is known to trigger internalization of ErbB2. This seems to involve several processes including increased membrane mobility of ErbB2 [44]. ERM proteins are important structural and regulatory components of the cortical membrane, therefore, we tested the effect of ERM inhibition on ErbB2 membrane mobility. For this purpose we performed FRAP experiments with GFP-ErbB2 in control cells and SKBR3 cells treated with the ERM inhibitor NSC668394 for 2 h or 4 h. Surprisingly, functional inhibition of ERM proteins significantly increased the time of fluorescence recovery and amount of the immobile fraction of ErbB2 (Figure 4A and 4B). In contrast to the EGFR receptor that undergoes ligand-activated internalization and is routed for subsequent lysosomal degradation [45, 46], ErbB2 is considered to be internalization impaired and recycled back to the plasma membrane [45, 47, 48]. Ubiquitination has been shown to be a regulatory mechanism for the downregulation of ErbB receptors [46]. However, it has been discussed that poor binding of ErbB2 to the E3 ubiquitin ligase c-Cbl might account for a low extent of ErbB2 ubiquitinylation [49, 50]. We performed PLA experiments to investigate the association of ErbB2 with c-Cbl upon ERM inhibition. In agreement with observations from others, we experienced only nominal association between ErbB2 and c-Cbl in untreated control cells. However, 20 min after treatment with NSC668394 we were able to observe a significant association between ErbB2 and c-Cbl (2x increased) and 4 h treatment resulted in a 25x increased interaction of ErbB2 with c-Cbl (Figure 4C and 4D). In line with this, we observed in another set of PLA experiments (Figure 4E) increased association of ErbB2 with ubiquitin after ERM inhibition. Furthermore, we confirmed the PLA data in co-IP experiments (Figure 4F). Thus, our data indicate that ERM-ErbB2 interaction may have an inhibitory effect on ErbB2 ubiquitination by preventing efficient binding of c-Cbl to ErbB2.

### ERM inactivation induces dynamin-dependent internalization of ErbB2 and subsequent sorting for degradation

**Figure 5:**
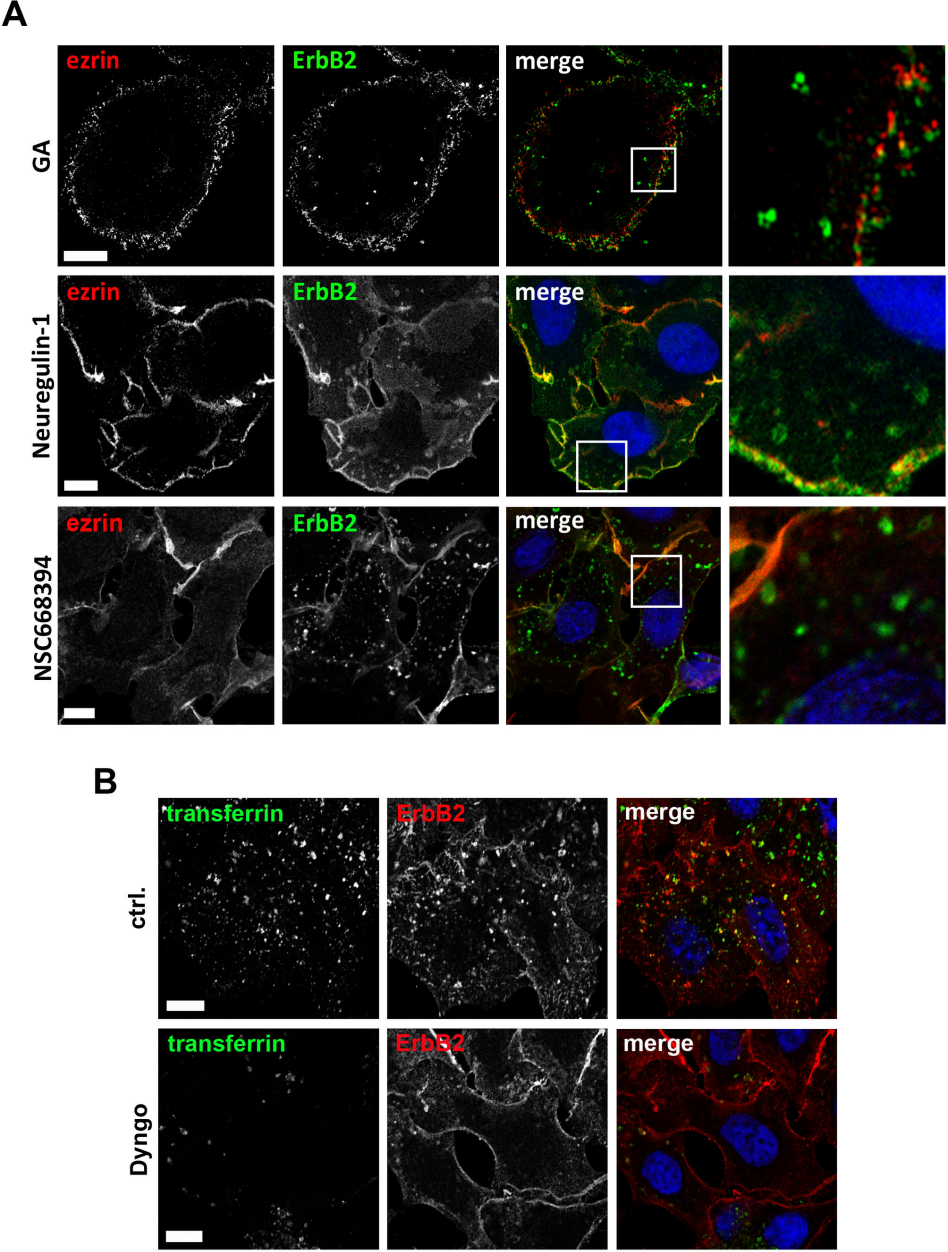
Microscopic analysis of ErbB2 internalization (**A**) Colocalization studies of ezrin with internalized ErbB2. ErbB2 internalization was induced by treatment of SKBR3 cells either for 2 h with 3 μm of geldanamycin (GA), 30 min with 1 nM neuregulin-1 or 2 h with 30 μM of NSC668394. Cells were fixed and stained for ErbB2 and ezrin. Analysis by 3D SIM (GA, NSC668493) or confocal microscopy (Neuregulin-1) shows a clear colocalization of ezrin with ErbB2 at the plasma membrane, whereas no colocalization was found with internalized ErbB2, independently of which component was used to trigger internalization. Scale bars: 6 μm (GA), 10 μm (neuregulin-1) and 8 μm (NSC668394). (**B**) Confocal microscopy of ErbB2 internalization upon ERM and dynamin inhibition. Cells were pretreated with 25 μM Dyngo 4a or DMSO (ctrl.) and subsequently incubated for 6 h with 30 μM NSC668394 before the cells were fixed and stained for ErbB2. 20 min prior to fixation, transferrin conjugated with Alexa488 was added to monitor dynamin dependent uptake. In control cells internalized ErbB2 was colocalized with internalized transferrin. However, ErbB2 uptake, was dramatically reduced or completely absent in cells with low or no uptake of transferrin upon treatment with Dyngo. Scale bars: 10 μm.

Next we wanted to characterize the process of ErbB2 internalization and subsequent trafficking mediated by ERM inhibition. In PLA experiments we demonstrated that geldanamycin treatment leads to reduced association of ErbB2 to ERM proteins (Figure 1C). By 3D SIM we observed that colocalization between ErbB2 and ERM proteins solely occurred at the plasma membrane, and that this interaction was disrupted after geldanamycin triggered ErbB2 internalization (Figure 5A, upper panel). Similarly, no intracellular colocalization was observed after treatment with neuregulin-1, an ErbB3 ligand that induces internalization of ErbB2-ErbB3 dimers (Figure 5A, middle panel). Treatment with NSC668394 inhibits phosphorylation and leads to dephosphorylation of ERM proteins without affecting total ERM levels. Despite the fact that we observed an increased staining of cytoplasmic ezrin after treatment of NSC668394, no colocalization of ezrin with internalized ErbB2 was found (Figure 5, lower panel). Hence, the intracellular fate of ErbB2 seems to be independent on its proximity to ERM proteins. However, this does not exclude the possibility that ERM proteins might be involved in the regulation of ErbB2 sorting (see discussion).

**Figure 6:**
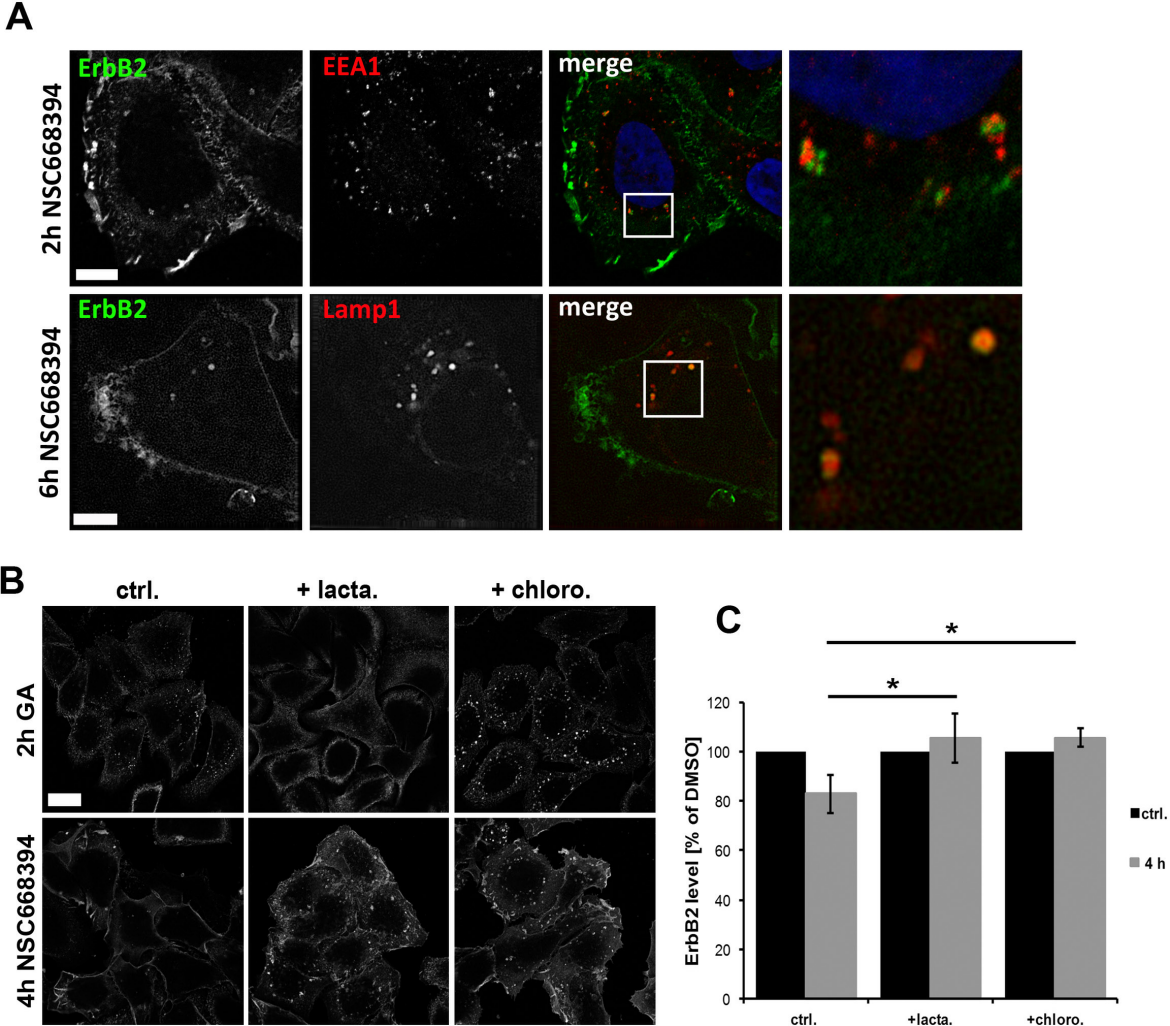
Analysis of intracellular sorting of ErbB2 by confocal microscopy (**A**) ErbB2 localization with EEA1 and Lamp1 after ERM inhibition. Cells were treated for 2 h or 6 h with NSC668394 and subsequently fixed and stained for ErbB2 and EEA1 or Lamp1. ERM inhibition leads to ErbB2 internalization and association to the endosomal marker EEA1 at early time points and association Lamp1-possitive vesicles at later time points. Scale bars: 10 μm. (**B**) Cells were pretreated for 2 h with 10 μM of lactacystin or chloroquine, followed by a 2 h treatment with 3 μM geldanamycin (GA) or 4 h with 30 μM of NSC668394. Afterwards, cells were fixed and stained for ErbB2. Lactacystin inhibits the uptake of ErbB2 triggered by GA but not by NSC668394. Chloroquine pretreatment leads in GA and NSC668394 treated cells to an intracellular accumulation of ErbB2 to vesicular structures. Scale bar: 10 μm. (**C**) Quantification of ErbB2 protein levels after lactacystin and chloroquine treatment. Cells were treated as described in (B), lysed and analyzed by SDS-PAGE. Treatment of cells with lactacystin or chloroquine inhibits degradation of internalized ErbB upon ERM inhibition by NSC668394. Data is shown as mean +/− SEM (*P < 0.05).

Induced internalization of ErbB2 by Hsp90 inhibition with geldanamycin is considered to be mediated by dynamin- and clathrin-dependent mechanisms [44, 51]. Therefore, we analyzed by which mechanisms ErbB2 is internalized upon functional inactivation of ERM proteins. SKBR3 cells were treated with NSC668394 with or without Dyngo 4a pretreatment. Dyngo has been shown to inhibit dynamin dependent internalization of transferrin, but to have no effect on dynamin-independent endocytosis of cholera toxin [52]. We observed either a strong reduction or complete inhibition of transferrin uptake in SKBR3 cells after Dyngo pretreatment. Also, we did not detect internalized ErbB2 in cells negative for transferrin uptake (Figure 5B). After 2 h treatment with NSC668394 internalized ErbB2 was associated with the endosomal marker EEA1, whereas after longer incubation times ErbB2 was sorted to Lamp1 positive vesicles (Figure 6A), corresponding to decreased ErbB2 levels after ERM depletion or inhibition (Figure 2B, 2E). It has been described that proteasomal activity is required for geldanamycin induced uptake of ErbB2, followed by endosomal sorting to lysosomes for degradation [44]. Accordingly, we observed no significant uptake of ErbB2 after treatment with geldanamycin in the presence of the proteasome inhibitor lactacystin. On the other hand, in presence of the lysosomal inhibitor chloroquine, ErbB2 was accumulated in intracellular vesicles upon geldanamycin treatment (Figure 6B). In contrast, lactacystin did not inhibit uptake of ErbB2 after ERM inhibition but led to intracellular ErbB2 accumulation in vesicular structures without effecting ErbB2 levels (Figure 6B, 6C). However, In the presence of chloroquine intracellular accumulation of ErbB2 was observed after NSC668394 treatment, similar to geldanamycin treated cells (Figure 6B, 6C). Treatment of SKBR3 cells with chloroquine or lactacystin and without inhibition of ERM proteins by NSC668394 did not affect the membrane localization of ErbB2 or pERM proteins (Supplementary Figure 4). Based on our results, we conclude that internalization and sorting of ErbB2 to endosomal vesicles, triggered by interference with ERM proteins, is independent of lysosomal or proteasomal activity. On the other hand, ErbB2 degradation can be blocked by lysosomal and proteasomal inhibition.

### Inhibition of ERM proteins results in dephosphorylation of ErbB2 and impaired Akt and Erk signaling

**Figure 7:**
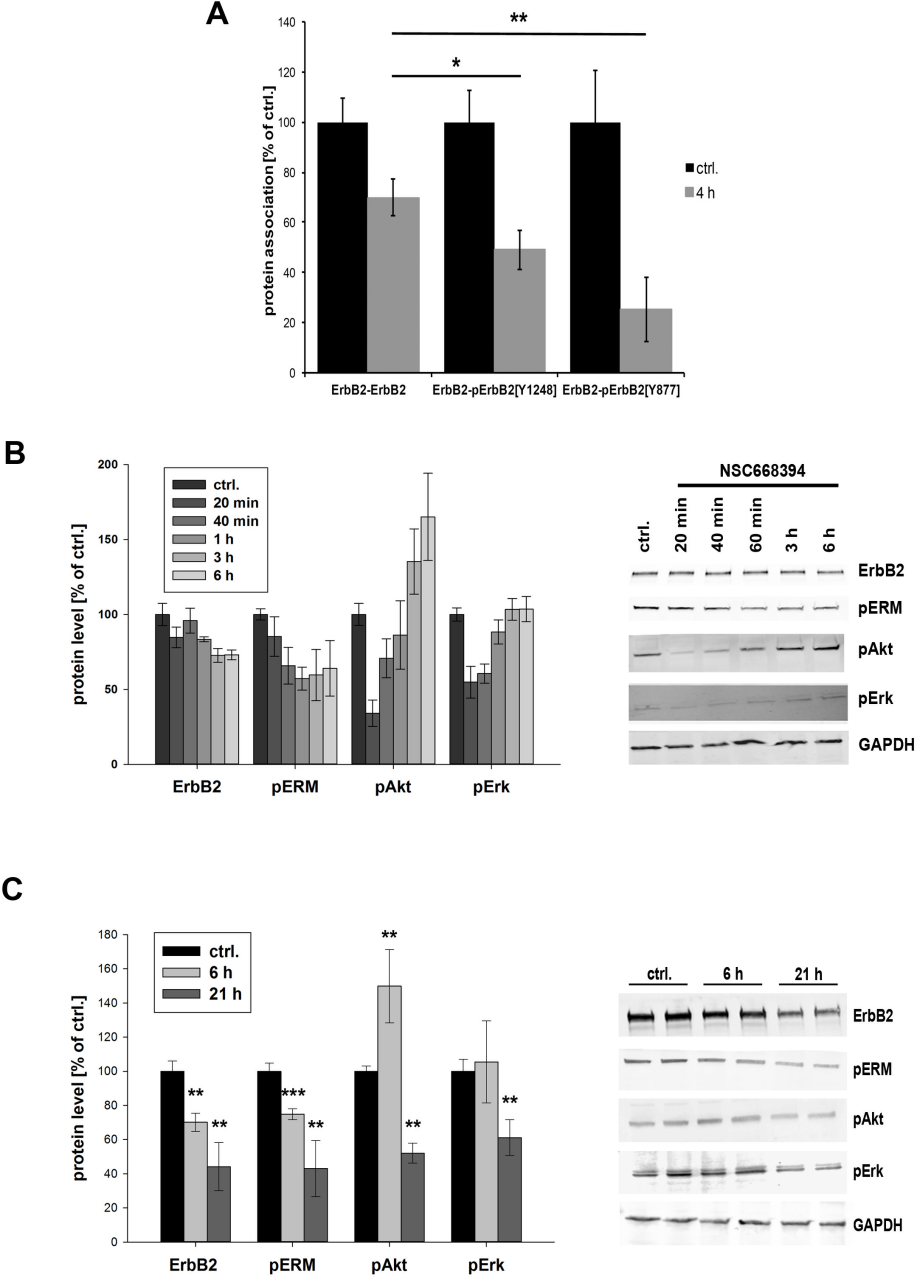
Phosphorylation status and downstream signaling of ErbB2 after ERM inhibition (**A**) Quantification of the phosphorylation of pErbB2^Y1248^ and pErbB^Y877^ by PLA. Cells have been treated with 30 μm NSC668493 or DMSO for 4 h and PLA experiments were performed on fixed cells. Inhibition of ERM proteins leads to a significantly stronger reduction in the levels of phosphorylated ErbB2^Y1248^ and ErbB2^Y877^ compared to total reduction of ErbB2. (**B, C**) Quantification of Western blot analysis of ErbB2, pERM, pAkt and pErk after treatment with NSC668394. SKBR3 cells were treated with 30 μM NSC668394 or DMSO (control), lysed after different time points and analyzed by SDS-PAGE. All data are shown as mean +/− SEM (*P < 0.05; **P < 0.01; ***P < 0.001).

After we demonstrated internalization and degradation of ErbB receptors in response to ERM depletion or inactivation, we finally wanted to analyze the effect on downstream signaling. The oncogenic potential of ErbB2-ErbB3 dimers is mediated by potent activation of signal transduction pathways, and receptor dimerization and trans-phosphorylation are fundamental for ErbB receptor signaling. Consequently, we analyzed in PLA experiments the phosphorylation status of ErbB2 after ERM inhibition (4 h NSC668493). In particular, we investigated the effects on the tyrosine phosphorylation of ErbB2^Tyr1248^ and ErbB2^Tyr877^. In order to separate effects on ErbB2 phosphorylation from ErbB2 degradation, we simultaneously monitored total ErbB2 levels. Consistent with Western blot data, we obtained 30% reduced ErbB2 level after 4 h treatment with NSC668394 (Figure 7A, ErbB2-ErbB2). Importantly, a significant stronger reduction on the phosphorylation of ErbB2^Y1248^ (50% reduction) and ErbB^Y877^ (75% reduction) was measured. Two of the main signaling pathways activated by ErbB2 and ErbB3, accountable for the strong oncogenic potential of this dimer, are the PI3K/Akt and the Ras/MAPK pathways. Thus, we analyzed the effect on the phosphorylation levels of Akt^Ser473^ and Erk1/2^Tyr202/Tyr204^. ERM inhibition led to a strong reduction of pAkt (65%) and pErk levels (45%) within 20 min (Figure 7B). However, within 3 h the levels of pErk were completely restored and pAkt levels were even 60% increased, compared to control cells. In order to investigate long time effects of ERM inhibition, we incubated SKBR3 cells with NSC668394 for 21 h. The long time incubation had no effect on cell morphology or cellular growth (Supplementary Figure 3C) but resulted in further decreased levels of ErbB2 (~60% reduction) and pERM (50%). Furthermore, the levels of pAkt and pErk were significantly reduced to approx. 55%, compared to control cells (Figure 7C). Thus, inhibition of ERM proteins results in impaired Akt and Erk signaling that can be separated into short- and long-term effects.

## DISCUSSION

Dependent on the biological context, ERM proteins have been described to be either functional redundant or to exhibit distinct biological functions. In our cellular system, ezrin and radixin showed an overlap in their function in ErbB2 regulation. We demonstrated that the presence and functional integrity of both proteins is important for a robust membrane localization of ErbB2 and ErbB3. ERM proteins show tissue- and cell-specific expression patterns and in contrast to many other cell lines, SKBR3 breast cancer cells do not express moesin. In line with this, moesin has been shown to be expressed in only 16% of all invasive ductal carcinoma and it is completely absent in several other breast cancer subtypes [53]. On the other hand, moesin has been considered as a potential marker for epithelial-mesenchymal transition (EMT) in breast and pancreatic cancer [53, 54] and it has been reported to be strongly upregulated in breast and basal breast carcinomas [53, 55, 56]. Thus, it cannot be excluded that also moesin might have a function in the regulation of ErbB receptor levels in other breast cancer cell lines or tissues. Moreover, the function of each single ERM protein in respect to ErbB regulation might be dependent on the cell- and tissue-specific context, and further investigations are needed to analyze this question.

In knockdown experiments and by rescue experiments we demonstrated that ezrin and radixin are required for the maintenance of cellular ErbB2 and ErbB3 levels. Since knockdown experiments are limited in respect of the evaluation of dynamic processes and rather favor endpoint studies, we further used the small molecule ERM inhibitor NSC668394. NSC668393 has been described to inhibit phosphorylation of ezrin T567 and also to inhibit threonine phosphorylation of radixin and moesin with slightly lower affinities [40]. The phosphorylation of a conserved N-terminal threonine residue, in combination with ERM binding to PI(4, 5) P_2_, is needed for functional activation or ERM proteins [57] Thus, NSC668394 treatment has been shown to prevent actin binding, resulting in several physiological effects, such as reduced cell migration, cell invasion and tumor growth [40]. Interestingly, in our experiments NSC668394 treatment had a more pronounced effect on ErbB2 degradation compared to knockdown experiments. This might be due to the fact that NSC668394 affects almost the entire cell population in a highly reproducible manner and that the selected concentration (30 μM) functionally inhibits all ERM proteins. Furthermore, long-term knockdown experiments are more likely to be counteracted by compensatory mechanisms.

Our data indicate the existence of a multiprotein complex consisting of ERM proteins, Ebp50, Hsp90, ErbB2 and ErbB3 in breast cancer cells. However, we have not been able to co-immunoprecipitate ERM proteins or Ebp50 together with ErbB2. It is known that the analysis of membrane-associated proteins can be technically demanding [58], and cell lysis might destroy the membrane environment required for the integrity of a ERM-Ebp50-ErbB2 complex. Nevertheless, depletion or functional inactivation of ERM proteins triggers the dissociation of this complex and in the context of ErbB2/3 regulation both ERM proteins seem to act in a similar manner. In PLA experiments depletion of radixin seems to have stronger effects than ezrin knockdown. However, this might be a result of differences in the knockdown efficiency, as we experienced the tendency of a more efficient depletion of radixin compared to ezrin. It seems very plausible that several other proteins or lipids may interact with or are essential components of this multprotein complex. For example, we described recently an involvement of the scaffolding flotillin proteins in membrane stabilization of ErbB2 and ErbB3 receptors [38, 39]. Furthermore, it has been reported earlier that hyaluronic acid stimulates the formation of a protein complex consisting of CD44, Hsp90, ErbB2 and ezrin, in TA3/St mammary carcinoma cells [20]. CD44 can bind to ErbB2 and a correlation between high levels of CD44 and ErbB2 and poor prognosis has been reported [20, 59]. However, only a small fraction of SKBR3 cells (less than 5%) expresses CD44 [60]. Therefore, it seems unlikely that CD44 is required for an ERM-ErbB2 interaction in this cell line. However, substantial levels of CD44 are found in other breast cancer cells [60]. Hence, CD44 might support the interaction of ERM-ErbB2 in those cell lines.

We demonstrate an inhibitory effect of ERM proteins on the binding of c-Cbl to ErbB2 dimers. In contrast to EGFR/ErbB1, ErbB2 shows impaired internalization and downregulation. Besides other mechanisms, reduced recruitment of the ubiquitin E3 ligase c-Cbl has been considered to be responsible for the internalization deficiency of ErbB2 [49]. E3 ligases are substantial components for mono- or poly-ubiquitination of lysine residues on different substrates, and ubiquitination has been demonstrated to induce internalization and downregulation of ErbB receptors [46, 61–63]. We obtained a negative correlation between ERM-ErbB2 association and c-Cbl-ErbB2 binding. This might be mediated either by direct competitive interaction between ERM and c-Cbl with ErbB receptors or via the ERM-mediated formation of a putative complex that interferes with c-Cbl to ErbB2 binding. Noteworthy, geldanamycin-induced internalization and downregulation of ErbB2 seem to be independent of c-Cbl [50].

The functional role of ezrin and radixin on ErbB receptors seems to be determined by their localization at the plasma membrane. ERM proteins, in particular activated pERM proteins, connect the plasma membrane with the actin cytoskeleton, and interference with the activation of ERM protein leads to changes in the rigidity of the plasma membrane [64]. ERM proteins are localized to lipid raft microdomains [65] and have been implicated in the clustering and activation of adhesion molecules and receptors [66–68]. In line with this, our data indicate that ERM proteins are required for the stabilization and integrity of ErbB receptor complexes at the plasma membrane. However, even though intracellular ErbB2 does not colocalize with ERM proteins, we cannot exclude the possibility that ERM proteins might be involved in regulation of ErbB2 sorting and recycling. Following this idea, enhanced ErbB2 degradation upon ERM depletion or inhibition might be then a consequence of impaired routing and trafficking. Indeed, ezrin has been shown to be important for the recycling of adrenergic receptors [10], and knockdown of moesin leads to decreased levels of Gb3 at the cell surface [12]. Furthermore, ERM proteins interact with the CORVET and HOPS complexes, and ERM proteins and the HOPS complex are required for the transition from early to late endosomes [9]. However, in HeLa cells depletion of components of the CORVET and HOPS complexes or ERM proteins results, in contrast to ErbB2, in the accumulation of EGFR in endosomal structures and delayed EGFR degradation [9]. Thus, based on the cellular context and the receptor type, ERM proteins seem to display different functions in the regulation of particular ErbB receptors.

In terms of ErbB2 internalization and downregulation, ERM proteins and Hsp90 seem to exhibit different mechanistic functions. It has been reported that geldanamycin-induced internalization of ErbB2 is dependent on proteasomal activity [44]. Also our results demonstrate that after geldanamycin treatment ErbB2 internalization can be prevented by the proteasome inhibitor lactacystin. However, upon ERM inhibition ErbB2 is found to be colocalized with the lysosomal marker Lamp1, and ErbB2 degradation can be inhibited by the lysosomal inhibitor chloroquine. This indicates that ErbB2 internalization and downregulation, triggered by ERM depletion/inactivation, depends on lysosomal activity. On the other hand, also the proteasomal inhibitor lactacystin prevented ErbB2 degradation after ERM inhibition, but did not affect ErbB2 internalization. This finding suggests that also proteasomal mechanisms might contribute to ErbB2 degradation. This is in line with other studies reporting proteasomal degradation of ErbB2 [69, 70]. Controversially, it has been shown in other studies that ErbB2 receptors are not a direct target for proteasomes but undergo lysosomal degradation [44, 71, 72]. Thus, the detailed role of proteasomal activity in the process of ErbB2 downregulation upon interference with ERM proteins remains to be clarified.

We observed impaired levels of pAkt and pErk upon ERM inhibition. Even though these effects seem to be specific to the inactivation of ezrin and radixin in breast cancer cells, it is likely that inactivation of ErbB-mediated signaling contributes only partially to this mechanism. Akt and Erk pathways can be triggered by a variety of mechanism and transmembrane proteins, and ERM proteins are known to interact and modulate several of these components. For instance, it has been demonstrated that there is a direct activation of Ras by ERM proteins [32–34] and Ras is a crucial component of the Erk signaling pathway. Thus, inactivation of ERM proteins can affect Akt and Erk signaling by different mechanisms and further studies are needed to analyze this regulation in detail.

Taken together, our data demonstrate a so far unknown link between ERM proteins and the localization and functional integrity of the ErbB2 receptor tyrosine kinase in breast cancer cells. We were able to provide novel mechanistic insights into how internalization impaired ErbB2 receptors are regulated by the ERM proteins ezrin and radixin. At the plasma membrane ErbB2 and ErbB3 seem to be associated with a multiprotein complex, including Hsp90, Ebp50 and ERM proteins. Interference with ERM proteins by depletion or functional inhibition leads to a disruption of this complex. Furthermore, binding of ErbB2 to c-Cbl and ErbB2 ubiquitination is induced, accompanied by ErbB2 dephosphorylation and internalization. Finally, as a consequence of ERM inhibition long-term signaling via the Ras/MAPK and PI3K/Akt pathways is strongly reduced and ErbB2 and ErbB3 levels are decreased. Thus, our findings provide novel insight into the mechanistic regulation of ErbB2 receptors, and new therapeutic strategies can be considered by specifically targeting ERM proteins in breast tumors.

## MATERIALS AND METHODS

### Antibodies and other materials

Antibodies used for immunoblotting: rabbit anti-ErbB2 (29D8, Cell Signaling), mouse anti-ErbB2 (Ab-17, Thermo Fisher Scientific), rabbit anti-ErbB3 (Ab-1328, Sigma Aldrich, MO), rabbit anti-Ebp50 (SLC9A3R1, Sigma Aldrich), rabbit anti-Ebp50 (A310, Cell Signaling), mouse anti-ezrin (E8897, Sigma Aldrich), rabbit anti-radixin (HPA000763, Sigma Aldrich), rabbit anti-pERM (3149, Cell Signaling), mouse anti-GAPDH (Ab-9484, Abcam), mouse anti-pErk1/2 (9106, Cell Signaling), rabbit anti-pAkt (4058, Cell Signaling), mouse anti-Ubiquitin (P4G7, Covance). Antibodies used for microscopical analysis: rabbit anti-ErbB2 (29D8, Cell Signaling), mouse anti-ErbB2 (9G6, Santa Cruz), rabbit anti-pERM (3149, Cell Signaling), rabbit anti-ezrin (3145, Cell Signaling), mouse anti-ezrin (E8897, Sigma Aldrich), rabbit anti-radixin (HPA000763, Sigma Aldrich), mouse anti-radixin (1E12, Novus Biologicals), rabbit anti-Ebp50 (SLC9A3R1, Sigma Aldrich), mouse anti-Hsp90 (sc-13119, Santa Cruz), mouse anti-c-Cbl (Upstate). HEPES, bovine serum albumin, geldanamycin, lactacystin, chloroquine and *n*-octylglucopyranoside were purchased from Sigma-Aldrich. The small molecule inhibitor NSC668394 was purchased from Calbiochem, and the dynamin-inhibitor Dyngo4a was purchased from Fisher Scientific. The plasmids encoding GFP-ErbB2 and CFP-ErbB3 were kind gifts from Dr. B. van Deurs (University of Copenhagen, Denmark).

### Cell culture

Human breast carcinoma SKBR3 cells (ATCC: HTB-30) and HeLa cells (ATCC: CCl-2) were grown in DMEM GlutaMAX^™^ (Invitrogen Life Technologies, CA), PC-3 prostate cancer cells (ATCC: CRL-1435) in DMEM/F-12 medium (Invitrogen), MCF7 cells (ATCC:HTB-22) in RPMI (Invitrogen Life Technologies, CA). All media were supplemented with 10% v/v fetal bovine serum (Sigma Aldrich, MO), 100 U/ml penicillin and 100 U/ml streptomycin (Invitrogen) for complete medium conditions. Cells were grown at 5% CO_2_ and seeded 24 h prior to experiments. For RNAi mediated knockdown, SKBR3 cells were seeded without serum and antibiotics in six-well plates with 2 ×10^5^ cells/well.

### Transfection of siRNA oligos

For siRNA transfection, ON-TARGETplus single and/or SMARTpool of four siRNA oligos were used to reduce unspecific off-target effects. Control non-targeting and ErbB2, ezrin and radixin targeting oligos were purchased from Thermo Fisher Scientific (Waltham, MA). Cells were transfected with 25 nM siRNA oligos by using Lipofectamine RNAiMAX transfection reagent (Invitrogen Life Technologies, CA) according to the manufacturer's protocol. The cells were transfected for 6 h and subsequently grown in complete growth medium for 3 additional days prior to experiments. For rescue experiments, cells were grown for ~50 h after siRNA transfection. Then they were transfected with 1 μg of plasmid carrying siRNA resistant genes (human ezrin siRNAr sequence; TCCGCAGAACTGAGCTCTG, designed and synthesized by DNA 2.0) per 6 μl FuGENE 6 transfection reagent (Roche) according to the manufacturer's protocol. Control cells were transfected with an empty mammalian expression vector. The cells were then grown for 15 h more prior to experimental procedures.

### Immunofluorescence, confocal microscopy, FRET, FRAP and super resolution imaging

Cells were grown on glass cover slips and fixed in 4% methanol-free paraformaldehyde (Polysciences) for 15 min at RT and permeabilized with 0.1% Triton X-100 in PBS for 2 min at RT, before blocking in 10% FCS for 1 h at RT. The samples were subsequently immunostained with primary antibodies in 10% FCS for 1 h at RT, followed by 45 min incubation with fluorophore-conjugated secondary antibodies (Jackson ImmunoResearch Laboratories) in 10% FCS. The samples were mounted in ProLong Gold with DAPI (Molecular Probes, Invitrogen Life Technologies, CA) and images were acquired by using confocal laser scanning microscopes (LSM 710 or LSM 780, Carl Zeiss) with a Plan-Apochromat 63x/1.4 Oil DIC objective. LSM Image Browser software (Carl Zeiss) or IMAGEJ software (National Institute of Health) were used for further image analysis and quantification of signal intensities.

For FRAP (fluorescence recovery after photobleaching) experiments SKBR-3 cells were plated on 60 mm plates with a cover glass inserted in the centre, allowed to grow for 24 h, and transfected with 1.0 μg ErbB2-GFP using Fugene 6 (Roche, Basel, Switzerland). After 16 hours the medium was substituted with HEPES buffer (20 mM HEPES pH 7.5, 140 mM NaCl, 2 mM CaCl_2_, 10 mM KCl, 1 mg/ml glucose), and the cells were studied at 37°C using a heated chamber on a Zeiss LSM 780 microscope using the FRAP module from the integrated Zen 2011 software. Studies of cells were performed after addition of DMSO (control condition) or treatment with 30 μM NSC668394 for different time periods. Fluorescence recovery of bleached regions was recorded for ~50 frames, and compensated for the loss of fluorescence due to scanning by comparing with unbleached regions. The data, corrected for background fluorescence and bleaching, were analyzed by non-linear regression and the exponential one-phase association model, by using the FRAP module in the Zeiss Zen 2011 software. The generated fluorescence recovery curves were used to obtain the percentage of maximum fluorescence recovery, which corresponds to the mobile fraction, and the half-time of maximum recovery (*t*_1/2_) values.

FRET (fluorescence resonance energy transfer) acceptor photobleaching was also performed with a Zeiss LSM 780 microscope as described before. For this purpose cells were fixed and permeabilized as described above and endogenous ErbB2 and ezrin were stained with Alexa488- and Alexa568-labeled antibodies, respectively. Alexa488 and Alexa568 fluorophores were excited by using 458 nm and 514 nm lasers, respectively. For FRET cells were imaged and bleached for 20 iterations with 100% laser intensity to 30% or less of the initial Alexa568 intensity. FRET efficiency was calculated as follows: FRET_eff_ (%) = (D_post_−D_pre_)/D_post_, where D_post_ is the fluorescence intensity of the donor after acceptor photo bleaching, and D_pre_ the fluorescence intensity of the donor before acceptor photo bleaching. The FRET efficiency, FRET_eff_, was determined after shift correction, cross-talk correction and correction for nonspecific bleaching of the donor fluorescence intensity (Alexa488) after acceptor bleaching.

3D SIM (structured illumination microscopy) imaging was performed on a DelatVision OMX V4 system (Applied Precision) equipped with an Olympus 60x numerical aperture (NA) 1.42 objective, cooled sCMOS cameras and 405, 488, 568 and 642 nm diode lasers. Z-stacks covering the whole cell were recorded with a Z-spacing of 125 nm. A total of 15 raw images (five phases, three rotations) per plane were collected and reconstructed by using SOFTWORX software (Applied Precision).

### Duolink – *in situ* proximity ligation assay (PLA)

*In situ* proximity ligation assay kit Duolink-II (Olink Bioscience) was used to analyze and quantify protein interactions [73–75]. The cells were washed in PBS, fixed and permeabilized as described before. Primary antibodies raised against ErbB2 and ErbB3 were used and the PLA reactions were performed as described in the manufacturer's instructions. All reagents used for the PLA assay were from Olink Bioscience. The samples were mounted with Duolink Mounting Medium (Olink Bioscience), images were acquired by confocal microscopy and analyzed with ImageJ software (National Institute of Health). For the negative controls and to test the specificity of the antibodies, only one antibody was used as a probe. Mitotic cells and cells from dense fields were excluded from the analysis.

### Western blotting

Cell lysates were prepared as described elsewhere [38]. Proteins were separated by SDS-PAGE on a 4–20% gradient gel (Protean-TGX, Bio-Rad) and blotted onto a PVDF membrane (Immobilon-FL, Millipore, MA). The membrane was blocked in 5% w/v BSA (Sigma) in PBS with 0.1% Tween-20 (PBS-T) before incubating with the primary antibody overnight at 4°C. After washing with PBS-T, IRDye infrared linked secondary antibodies (LI-COR Biosciences) were used according to the manufacturer's manual. The bands were detected by Odyssey Infrared Imaging System (LI-COR Biosciences) and quantified using ImageJ (NIH). Levels of GAPDH were used as internal loading controls.

### Immunoprecipitation

For immunoprecipitation experiments ErbB2 antibodies (29D8, Cell Signaling) were conjugated with Dynabeads^®^ Protein G as described in the manufacturer's instructions (ThermoFisher Scientific). A normal IgG was used as a negative control for IP. Cells were treated with 3 μM geldanamycin (for 30 min and 60 min) or treated with 30 μM NSC668304 (for 1 h or 2 h), subsequently lysed as described above and incubated for incubated for 15 min at RT with the ErbB2-conjungated Dynabeads. After washing and processing of the probes as described in the manufacturer's protocol, the samples were analyzed by SDS-Page and ErbB2-ubiquitin conjugates were probed with an ubiquitin-specific mouse antibody (P4G7, Covance).

### Statistics

Student's *t*-test or Mann-Whitney Rank Sum test was used to calculate the *P*-value for all experiments. A *P*-value of 0.05 or less was considered to be statistically significant. A minimum of 3 experiments were performed and quantification of the data were given as mean ± SEM. For Duolink experiments, a minimum of 50 cells per condition and experiment were analyzed.

## SUPPLEMENTARY MATERIALS FIGURES


