# Supplementary material for: Regulation of ErbB2 localization and function in breast cancer cells by ERM proteins
Source: Oncotarget. 2016 Mar 23;7(18):25443–60. doi: 10.18632/oncotarget.8327 (PMC5041916; doi:10.18632/oncotarget.8327)
Supplement: Supplementary file 1 [file oncotarget-07-25443-s001.pdf]

Regulation of ErbB2 localization and function in breast cancer cells by ERM proteins

Supplementary Materials

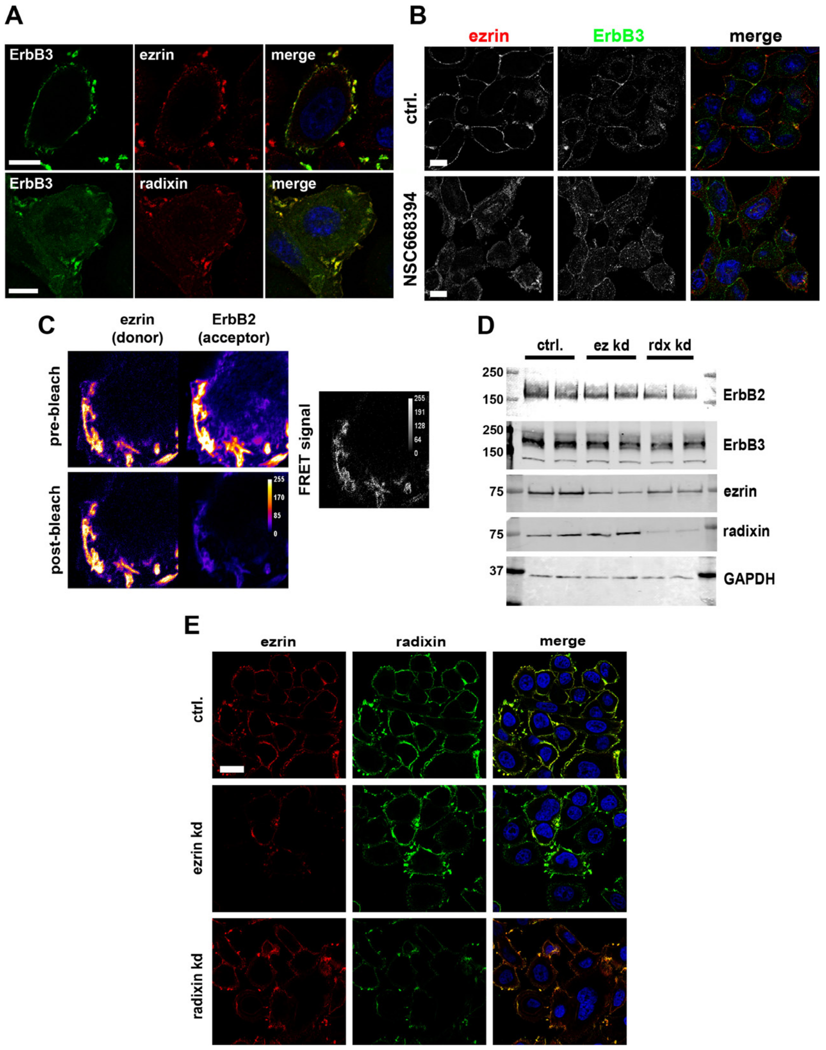

Supplementary Figure S1: Colocalization and association of ERM proteins with ErbB2 and ErbB3. (**A**) Colocalization of ERM proteins and ErbB3 in SKBR3 cells. SKBR3 cells have been transfected with CFP-ErbB3 for 16 h, afterwards cells were fixed and permeabilized and ezrin and radixin were stained by specific antibodies. Similar to ErbB2, ERM proteins show a clear colocalization with ErbB3 (single confocal planes). Scale bars: 10 µm. (**B**) Localization of endogenous ErbB3 upon ERM inhibition. Cells were treated with DMSO (ctrl.) or NSC668394 for 4 h, fixed and permeabilized. Ezrin and ErbB3 were stained by specific antibodies. Upon ERM inhibition ErbB3 shows an increased cytoplasmic localization (single confocal planes). Scale bars: 10 µm. (**C**) FRET acceptor photobleaching. SKBR3 cells were fixed, permeabilized and stained for ezrin and ErbB2 by specific antibodies. Ezrin was labeled with Alexa488 (donor) and ErbB2 with Alexa 567. Pictures were taken before acceptor bleaching (pre-bleach) and after bleaching (post-bleach). The signal for ErbB2 is strongly reduced after bleaching, whereas the signal for ezrin was increased due to unquenching. The FRET signal shows the increase of the donor signal after bleaching. The intensity values are shown in the calibration bar. Scale bar: 5 µm. (**D**) Western blot analysis of SKBR3 cells depleted for ezrin or radixin. Cells were treated with specific siRNA and incubated for additional 72 h. Cell lysates were analyzed by SDS-PAGE and probed with indicated antibodies. (**E**) Confocal microscopy of ezrin and radixin knockdown cells (single confocal planes). SKBR3 cells were depleted for ezrin or radixin and endogenous ERM were detected by specific antibodies levels in fixed and permeabilized cells. Scale bar: 10 µm.

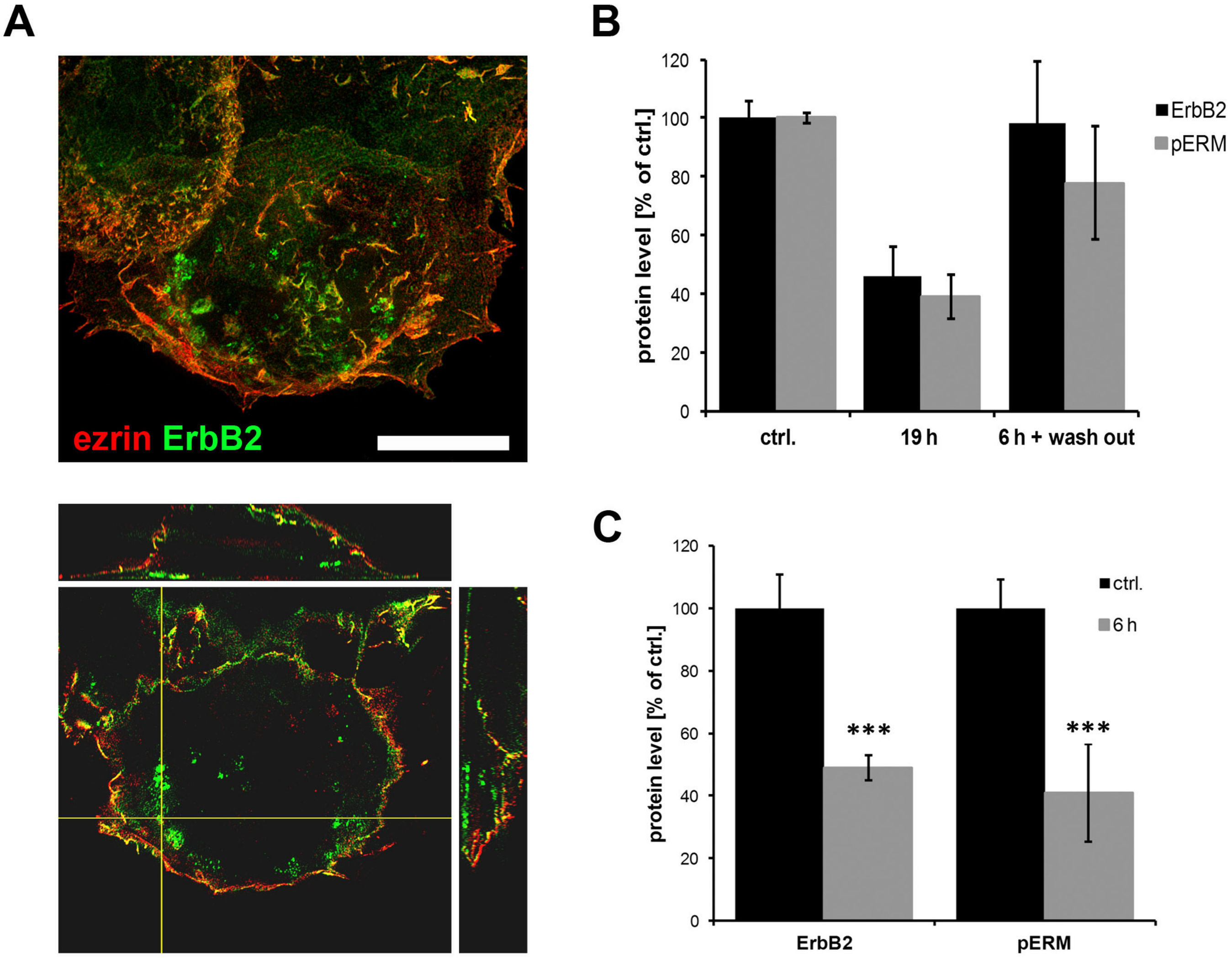

Supplementary Figure S2: Downregulation of ErbB2 by inhibition of ERM proteins. (**A**) 3D-SIM of cells after treatment with NSC668394. Cells were treated for 4 h with NSC668394, fixed, permeabilized and stained for ezrin and ErbB2. Upper panel shows the max. projection of the whole cell volume. The lower panel represents an orthogonal view of the same cell. (**B**) Wash out of NSC668493 restores pERM and ErbB2 levels. SKBR3 cells incubated for 19 h with NSC668394 show ~60% reduced levels of ErbB2 and pERM levels. Cells where NSC668394 has been replaced by fresh medium and incubated for additional 13 h, show a clear regeneration of pERM and ErbB2 protein levels. (**C**) Quantification of Western blot analysis of ErbB2 and pERM levels in MCF7 cells after treatment with NSC668394 for 6 h. Data is Figure represented as mean +/− SEM (****P* < 0.001).

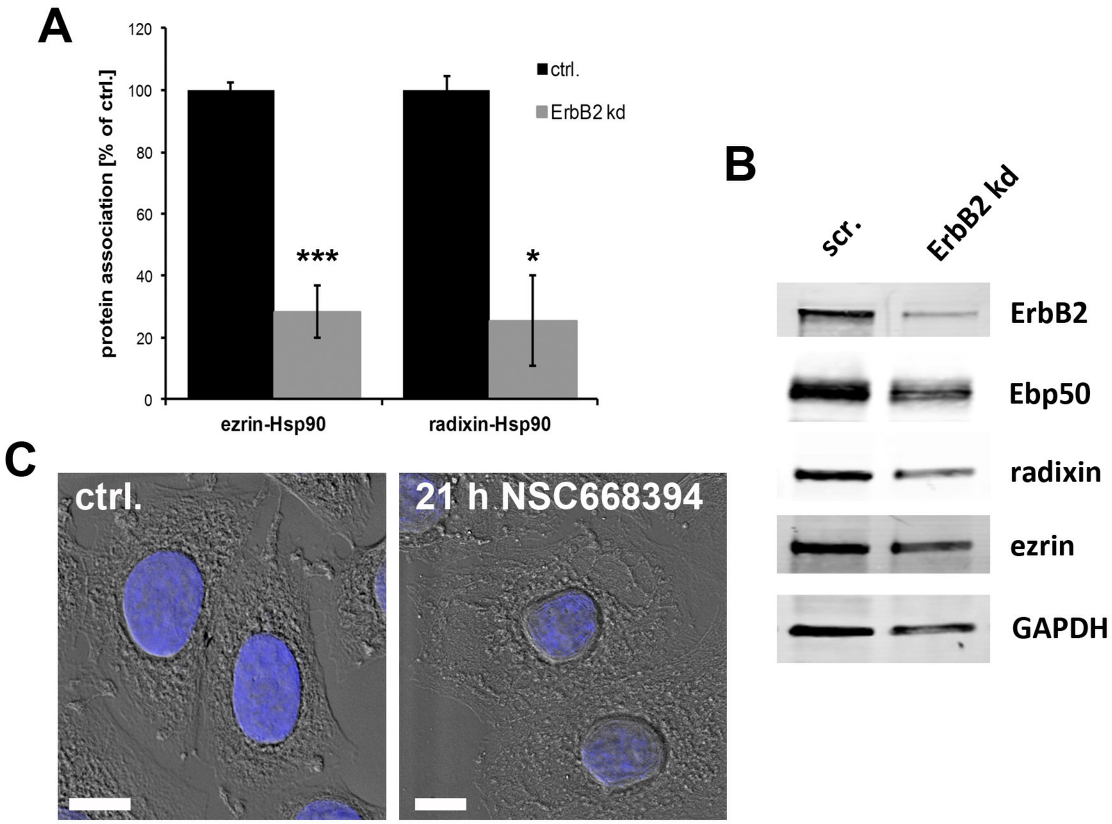

Supplementary Figure S3: Effect on ErbB2 knockdown on ERM-Hsp90 association and protein levels. (**A**) Quantification of ERM-Hsp90 association by PLA experiments. Knockdown of ErbB2 leads to strong dissociation of ERM proteins from Hsp90. The association of ezrin or radixin to Hsp90 is 70% reduced after knockdown of ErbB2. (**B**) ErbB2 knockdown has no specific effect on ERM and Ebp50 protein levels. Western blot of SKBR3 cells depleted for ErbB2 showed slightly reduced total protein levels but no specific effect on Ebp50, ezrin or radixin compared to control cells transfected with non-targeting control siRNA (scr.). GAPDH was used a loading control. (**C**) Cell morphology after ERM inhibition. DIC pictures of fixed cells after treatment with DMSO (ctrl.) or NSC668394 for 21 h. DAPI staining of nuclei in blue. Scale bars: 10 µm.

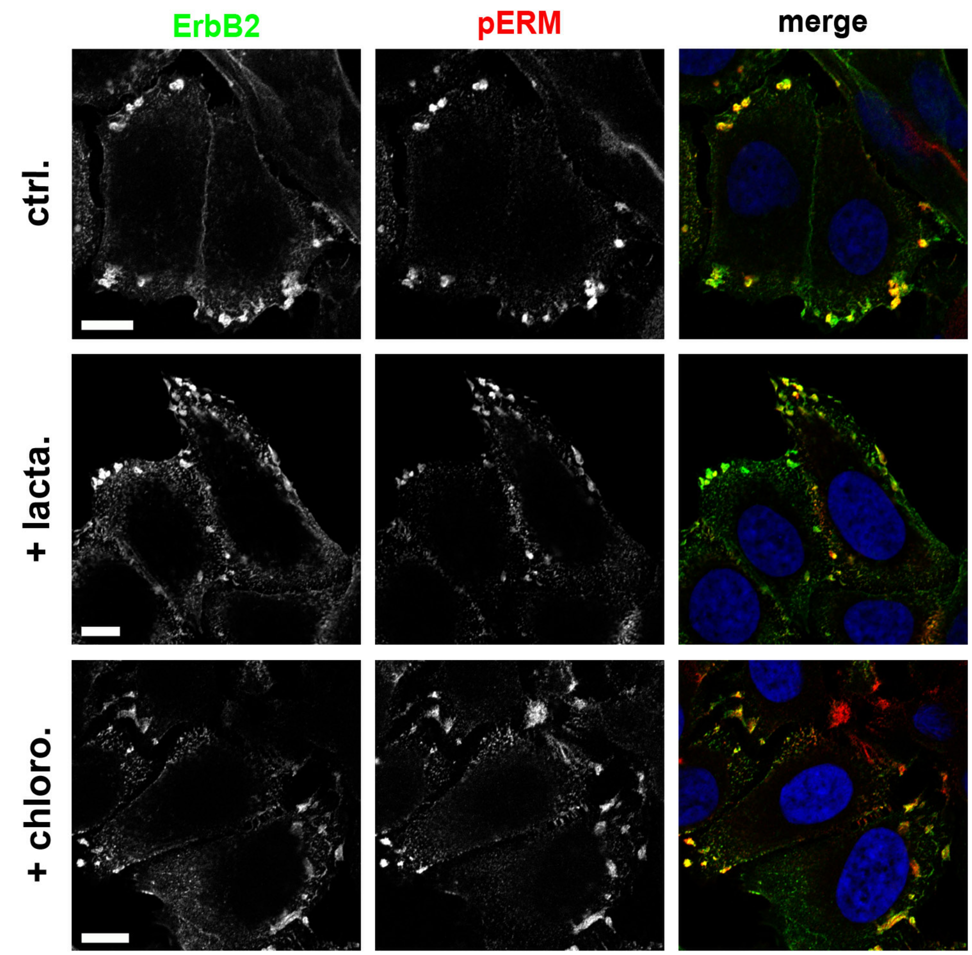

Supplementary Figure S4: ErbB2 localization after lysosomal and proteasomal inhibition. SKBR3 cells were treated for 6h with 10 µM of lactacystin or chloroquine, and control cells were left untreated. Afterwards, cells were fixed and stained for ErbB2 and pERM and analyzed by confocal microscopy. Neither treatment with lactacystin nor chloroquine induced obvious changes in the membrane localization of ErbB2 and pERM proteins. Scale bars: 10 µm.
